# Impact of community-based health insurance on health services utilisation among vulnerable households in Amhara region, Ethiopia

**DOI:** 10.1186/s12913-023-09024-3

**Published:** 2023-01-19

**Authors:** Essa Chanie Mussa, Tia Palermo, Gustavo Angeles, Martha Kibur, Frank Otchere, Amhara ISNP Evaluation Team, Maja Gavrilovic, Elsa Valli, Jennifer Waidler, Sarah Quiñones, Ana Gabriela Guerrero Serdan, Vincenzo Vinci, Lisa-Marie Ouedraogo, Getachew Berhanu Kebede, Getinet Tadele, Sewareg Adamu, Teketel Abebe, Yenenesh Tadesse, Feredu Nega, Mesay Kebede, Fekadu Muluye, Alene Matsentu, Daniel Aklilu

**Affiliations:** 1 UNICEF Office of Research—Innocenti, Via Degli Alfani 58, Florence, 50121 Italy; 2 Department of Agricultural Economics, University of Gondar, Gondar, Ethiopia; 3 Department of Epidemiology and Environmental Health, University at Buffalo (State University of New York), Buffalo, NY 14214-8001 USA; 4 Department of Maternal and Child Health, UNC Gillings School of Global Public Health, University of North Carolina at Chapel Hill, Chapel Hill, NC 27516 USA; 5 UNICEF Country Office of Ethiopia, Box 1169, Addis Ababa, Ethiopia

**Keywords:** Cash transfers, Community-based health insurance, Integrated safety net programme, Health services utilisation, Ethiopia, Africa

## Abstract

**Background:**

Ethiopia piloted community-based health insurance in 2011, and as of 2019, the programme was operating in 770 districts nationwide, covering approximately 7 million households. Enrolment in participating districts reached 50%, holding promise to achieve the goal of Universal Health Coverage in the country. Despite the government’s efforts to expand community-based health insurance to all districts, evidence is lacking on how enrolment in the programme nudges health seeking behaviour among the most vulnerable rural households. This study aims to examine the effect of community-based health insurance enrolment among the most vulnerable and extremely poor households participating in Ethiopia’s Productive Safety Net Programme on the utilisation of healthcare services in the Amhara region.

**Methods:**

Data for this study came from Amhara pilot integrated safety net programme baseline survey in Ethiopia and were collected between December 2018 and February 2019 from 5,398 households. We used propensity score matching method to estimate the impacts of enrolment in community-based health insurance on outpatient, maternal, and child preventive and curative healthcare services utilisation.

**Results:**

Results show that membership in community-based health insurance increases the probabilities of visiting health facilities for curative care in the past month by 8.2 percentage points (95% CI 5.3 to 11.1), seeking care from a health professional by 8.4 percentage points (95% CI 5.5 to 11.3), and visiting a health facility to seek any medical assistance for illness and check-ups in the past 12 months by 13.9 percentage points (95% CI 10.5 to 17.4). Insurance also increases the annual household per capita health facility visits by 0.84 (95% CI 0.64 to 1.04). However, we find no significant effects of community-based health insurance membership on utilisation of maternal and child healthcare services.

**Conclusions:**

Findings that community-based health insurance increased outpatient services utilisation implies that it could also contribute towards universal health coverage and health equity in rural and informal sectors. The absence of significant effects on maternal and child healthcare services may be due to the free availability of such services for everyone at the public health facilities, regardless of insurance membership. Outpatient services use among insured households is still not universal, and understanding of the barriers to use, including supply-side constraints, will help improve universal health coverage.

**Supplementary Information:**

The online version contains supplementary material available at 10.1186/s12913-023-09024-3.

## Background

United Nations (UN) member countries have committed to achieving universal health care coverage (UHC) by 2030 under the Sustainable Development Goals (SDGs). This target, under SDG 3 (Ensure healthy lives and promote wellbeing for all at all ages), is motivated by recognition of the need for and access to quality essential healthcare services, medicines, and vaccines for all people and to facilitate financial risk protection [1]. However, past studies show gaps towards this goal. For example, in 2013, the median proportion of births attended by a skilled health worker across 75 low and middle-income countries (LMICs) was only 62% [2] and, in 2013 about 400 million people globally lacked access to at least one of the essential healthcare services including family planning, receiving at least four antenatal care (ANC) visits, receiving 3 doses of diphtheria, tetanus, and pertussis (DTP) vaccine, antiretroviral therapy, tuberculosis treatment and children sleeping under insecticide-treated bed nets (ITBNs) [3]. Further, during 2014–2020, only 41% and 52% of children in western and central Africa and eastern and southern Africa, respectively, with symptoms of acute respiratory infection were taken to a health facility [4]. To address these and related gaps, many low-income countries are giving increasing attention to health insurance in efforts to increase health care utilisation and the attainment of UHC [5–7]. In this regard, Community-Based Health Insurance (CBHI) has been used as one of the important tools to expand access to healthcare services by the poorest and most vulnerable groups [8, 9].

As part of the country’s efforts to strengthen the supply and increase the demand for health services, Ethiopia implemented a series of health sector development plans in the past two decades, including the last five-year Health Sector Development Programme (HSDP) (2010/11 – 2014/15) [10] and the first Health Sector Transformation Plan (HSTP I) (2015/16—2019/20) [11]. The government of Ethiopia also offers all the services delivered at the health posts as well as maternal (including family planning, antenatal care, delivery, and postnatal care) and child-related health services such as child immunizations delivered at all public health facilities free of charge regardless of socio-economic status [12, 13]. The country also invested heavily in expanding health facilities and development of health professionals to deliver quality healthcare services [14, 15]. The government of Ethiopia also provides a healthcare fee waiver for about 2 million individuals (approximately 10% of the population living below the national poverty line) annually to get healthcare services at no cost [11]. Partly to gradually and systematically replace the healthcare fee waiver scheme [16], the country has also been implementing CBHI since 2011 to further expand access to essential health care services and increase health seeking behaviour of individuals while protecting households against catastrophic health expenditures. Accordingly, some improvements have been registered. For example, the proportion of fully immunised under-one children increased from 24% in 2011 to 39% in 2016 [17] and delivery at a health facility increased from 26% in 2016 to 48% in 2019 [18]. Despite these achievements, critical gaps remain. To mention a few, in 2016, only 31% of under-five children with symptoms of acute respiratory illness (ARI) and 35% of children with a fever sought services from a health care facility or provider [17]. Further, the most recent demographic and health survey (DHS) also reported that only 43% of women had at least four ANC visits during their previous pregnancy and 34% of women received post-natal care (PNC) within two days period [18]. In addition, although the per capita outpatient department visits increased from 0.3 in 2013/14 [14] to 0.9 in 2019, it is still far below the WHO recommendation of 2.5 per capita annual visits [19].

Against the backdrop of the government’s commitments but substantial gaps in some key health outcome indicators, this study examines whether CBHI increases health services utilisation among some of the most vulnerable rural households who participate in the government poverty-targeted social protection programme - the Productive Safety Net Programme (PSNP). Previous studies from LMICs on the impacts of CBHI on health services utilisation find mixed evidence. Various studies show that CBHI enrolment is linked to increased preventive healthcare utilisation such as sleeping under insecticide-treated bed nets or vaccination for children [20, 21], utilisation of outpatients health care [22–26], use of some maternal health care services [27], and better self-reported health and higher perceived quality of services [23–25]. In contrast to this, some other studies reported insignificant effects of CBHI membership on the utilisation of inpatient health services [20, 24, 26].

In Ethiopia in particular, studies to date have been conducted on the impacts of CBHI on health service utilisation using data collected during CBHI’s pilot phase [24, 25] and using small-scale household surveys [28, 29]. Past studies generally show that CBHI enrolment is likely to increase healthcare utilisation, decrease costs per visit, better self-reported health, and higher perceived quality of services [23–25]. More specifically, Demissie and Negeri [28] find that membership in CBHI is associated with a three-fold increase in the utilisation of outpatient healthcare services in southern Ethiopia. Elsewhere in Ethiopia, Tilahun et al*.* [29] also find that being a member to CBHI increases healthcare utilisation approximately by 25.2 percentage points. A study by Atnafu et al*.* [29] also shows that households who were enrolled in CBHI were more likely to use healthcare services than households who were not enrolled. Mebratie et al*.* [25] find that utilisation of outpatient services in public health facilities increases by 30–41% and the frequency of visits by 45–64% due to membership in CBHI. Similarly, Shigute et al*.* [30] also reported that CBHI nudges the probability of using modern healthcare services (visiting a modern health care facility for outpatient care services) and the number of visits to modern health facilities among adult members in the aggregated sample. They find a larger impact of CBHI on outpatient health services utilisations for the PSNP sub-sample compared to the pooled sample. Utilisation of child curative care services for an illness in the past 4 weeks also increased due to enrolment in CBHI [31].

Existing studies in Ethiopia have focused on the general population, regardless of households’ participation in other social protection programmes such as Ethiopia’s flagship social protection program, the PSNP, while in the current study, we focus particularly on PSNP-participating households. Thus, CBHI enrolled households in our study could be members of two large-scale social protection programmes: CBHI and PSNP, thereby giving important policy insights on how membership in CBHI and PSNP affects households’ health seeking behaviour. In this regard, we aim to contribute to the scant literature by focusing entirely on PSNP beneficiary households. From a policy perspective, our study gives new evidence to better understand how the integration of social assistance programmes can affect the utilisation of health services among the most vulnerable population. Further, while previous studies examined limited healthcare utilisation indicators, mainly outpatient visits and inpatient services, our study considered several healthcare services, categorized under outpatient, maternal, and child preventive and curative services.

## Methods

### Policy context

In 2011, the government of Ethiopia piloted CBHI in 13 rural districts (covering about 1.6 million people) targeting rural households and people working in the informal sector. This was scaled up to 161 districts after three years of piloting [32]. As of 2019, the programme covered 7 million households residing in 770 districts throughout the country (i.e., 75% district coverage nationwide). CBHI is currently operating in all regions and Addis Ababa except Somali, Gambella, and Dire Dawa. In the programme districts, 50% of eligible households are currently enrolled and the programme has an 82% renewal rate [33]. Nevertheless, the national level enrolment is still below the target set by the government: 80% of household enrolment and 80% coverage of districts by 2020 [11].

Enrolment in CBHI is conducted voluntarily. The programme uses the core principles of risk-sharing, a community-based decision-making process, and community support. Enrolment is conducted at the household level and all rural households in the district, excluding those formally employed, can join the programme.

The CBHI is a yearly contractual agreement with advance premium payments by the members, and all renewals and new member registrations are conducted for a period of up to three months every year. Currently, the programme has two member types – self-paying and indigent members. The regional and district governments jointly fund the enrolment premiums of indigent households such as the permanent direct support (PDS) clients in the productive safety net programme. For paying members, annual premiums are set based on household sizes. In 2019, the premiums were ETB 240 (USD 8.6) for 1 to 5 member households, ETB 290 (USD 10.4) for 6–7 member households, and ETB 340 (USD 12.2) for households with 8 or more members.

The benefit package of CBHI programme includes all outpatient and inpatient services available in health centres, treatment for cancer, dialysis and organ transplant for renal failure, treatment of major trauma, intensive care unit, hip and knee replacement, and major burns [32]. Services sought at primary, general, and referral public hospitals are also covered following appropriate referral procedures [34]. All services must be sought from public healthcare facilities with contractual agreements with the district CBHI office. CBHI does not cover costs related to tooth implantation, eyeglasses for ophthalmic cases, cosmetic procedures [32], aesthetic surgery, infertility treatment, and organ transplants (except renal, heart, and bone marrow) [34].

Ethiopia’s government also enacted its flagship poverty-targeted social protection programme, the rural Productive Safety Net Programme (PSNP), in 2005. About 85% of the programme beneficiaries are required to work on labour-intensive Public Works (PW) for payments while the other 15%, called the Permanent Direct Support (PDS) clients, who lack labour to participate in public works, receive unconditional cash and/ or food transfers [35]. To integrate various social protection programmes, the government endorsed its National Social Protection Policy (NSPP) in 2014 and launched the National Social Protection Strategy (NSPS) in 2016. However, Hirvonen et al*.* [36] found limited linkages between these large-scale social protection programmes. The Integrated Safety Net Programme (ISNP) is designed to address this gap. This pilot project, with the technical support from the United Nations Children’s Fund (UNICEF) Ethiopia country office (ECO), aimed to reinforce the linkages between the PSNP and CBHI and leverage the impacts of PSNP to reduce poverty and improve the multidimensional well-being of PSNP-participating households. The efforts to integrate the social protection programmes also assume that increasing coverage of CBHI among PSNP-participating households will increase their health services utilisation and improve health outcomes. The ISNP was launched in 2019 [37].

### Study setting

This study used cross-sectional data from the ISNP impact evaluation baseline survey in Amhara region, Ethiopia [38]. The ISNP evaluation is being carried out in 4 rural districts of Amhara region, namely, Libo Kemkem and Dewa Chefa as treatment districts and Ebinat and Artuma Fursi as comparison districts. Households in treatment districts receive additional ('plus') interventions on top of PSNP cash transfers including facilitation to CBHI enrolment, nutrition information through behavioural change communication (BCC) sessions, and case management through social workers and community care coalitions, while those in comparison districts do not get these plus components. While the treatment districts were selected purposively based on the availabilities of CBHI in the district, UNICEF ECO nutrition interventions and linkages to other UNICEF interventions, districts’ accessibility and practicality for UNICEF ECO support, comparisons districts were selected based on their similarities with treatment districts in socio-demographic, health service supply, programme organization, culture/ ethnicity, and ecological characteristics. Thus, the treatment and their respective comparison districts are geographically close and similar culturally and economically. The trial is registered on November 5, 2018, in the Pan African Clinical Trial Registry with trial registration ID—PACTR201902876946874. More information about the overall ISNP evaluation and interventions can be found in the online Additional file 1: Appendix 1. However, in the current study, we do not examine programme impacts of the INSP, but rather we use the baseline data to examine the effects of CBHI on health services utilisation.

### Study design and participants

The ISNP evaluation employed a mixed-method study approach. However, this study used the quantitative data generated through household, community, and health facility surveys. Households eligible for the survey include all PSNP-participating rural households in the four districts. The sample size was determined using power calculation based on estimates of baseline means and the expected impacts of indicators. The indicators included individuals’ health services utilisations during the last month, visiting or consultation of a health service provider in the last 4 months, enrolment in CBHI, child nutrition and preventive health indicators, and mothers receiving antenatal care from a skilled provider during the last pregnancy. For each indicator, the sample size was calculated to detect a desired change of delta (δ) with minimum power of 80% under the assumption of simple random sampling and zero non-response rate. Accordingly, a target sample size of 5,400 households was decided, of which 5,398 were interviewed.

### Data collection

The household questionnaire was designed to capture a broad range of information both at the individual and household levels such as demographics, educational attainment, health status and utilisation, PSNP participation, asset ownership, food security, and dwelling characteristics. Questionnaire items were drawn from previously implemented questionnaires and validated measures, including from the Transfer Project and other surveys implemented in Ethiopia and Eastern Africa (see Online Additional file 1: Appendix 6 for details about the variables) [39]. Some sections draw directly from other standard surveys such as the Multiple Indicator Cluster Survey (MICS), and instruments were tested in Ethiopia during piloting of the questionnaire at data collection trainings and then adapted as needed. A proxy female respondent from each household (priority was given to the main woman of the household or caregivers of children) was interviewed. Enumerators used electronic tablets installed with programmed survey (Survey Solutions) tools to input data and interviews were administered face-to-face in local languages (Amharic in Libo Kemkem and Ebinat districts and Afan Oromo in Dewa Chefa and Artuma Fursi districts). Baseline data collection was conducted between December 2018 and February 2019.

For the community surveys (one per kebele (village) – the lowest administrative level in Ethiopia), community leaders and knowledgeable individuals in each sector were interviewed. Health care workers or facility administrators were interviewed for the health facility surveys on the facility characteristics/ infrastructure, personnel, and supplies. Data were also collected from official logbooks in all government health care facilities in study communities.

### Measurements

The CBHI enrolment is the treatment variable. It is defined as holding a currently valid or renewed CBHI card, which is determined at the household level (i.e., once a household enrols all members of that household are automatically enrolled, except for additional fees required for adult children). Households were coded 1 if they were currently enrolled in CBHI and 0 if they were not enrolled.

Outcomes of interest included primary preventive health services (child received all vaccinations (BCG, three doses of Polio vaccine, three doses of Pentavalent vaccine, and Measles) and mother received at least four ANC services and PNC visits in the past 12 months for children, children sleeping under long-lasting ITBNs, delivery at a health facility, births attended by skilled professionals, and children given deworming in the past 6 months). Child curative services considered in the study included health facility visits to seek treatment for child illness last month and any health facility visits for children in the last 12 months). We also considered outpatient services by members including any facility visits for curative services for illness in the past one month and if they also sought curative cares from health professionals. Data were collected on members’ facility visits to seek medical assistance for an illness and check-ups from health facilities in the past 12 months, and, if yes, the number of visits to a health facility for illness by all members in the household in the past 12 months. We excluded behaviours related to seeking medications over the counter and alternative care services from our analyses. Since CBHI enrolment is at the household level, we aggregated all outcomes at the household level. Accordingly, for outcomes observed at the individual level (adult members and under-five children), we consider the household as a service user if at least one member utilized the service.

Covariate selection for the propensity score matching analysis was guided by the principles that: 1) omission of important variables could seriously increase bias in estimates [40, 41], 2) only those variables that simultaneously influence participation decision and the outcome should be included [42], and 3) selected covariates should not be affected by participation decision, that is variables should either be time-invariant or measured before participation took place [42]. Accordingly, we used previous studies, economic theory, and study context to select covariates. Household head-related factors included sex, age, current marital status, disability, and literacy status. The household-level factors were wealth status, number of household members by age, access to improved water during winter (the dry season in Ethiopia), whether the household worried about food in the last 4 weeks, number of food insecurity months in the last 12 months, having outstanding debt, drought in the last 12 months, total annual income received from PSNP, number of ill household members in the last month, and indices on perceptions and understandings about CBHI generated using Factor Analysis (see Online Additional file 1: Appendix 6 for details). The study also controlled for community and health facility-related characteristics including distance from the village to the nearest health centre (kilometres), distance from the village to the nearest health facility with a doctor (kilometres), whether the nearest health facility admits people covered with CBHI, number of years the village has been in PSNP, and village distance from district capital (kilometres). Estimations also included district fixed effects.

### Ethical considerations

The study was approved by the Amhara Public Health Institute (APHI) Research Ethics Review Committee (Reference Number HRTT—03/192/2018). Enumerators received instruction during data collection training about ethical data collection, informed consent, and referral services and procedures. Informed consent was obtained from all survey participants to use their anonymised information.

This study does not involve patients. An inception workshop was conducted to select treatment districts using several social and economic indicators. Findings from the baseline data collection were disseminated in a consultative workshop conducted in August 2019 with the Amhara region and district administrators, Amhara Public Health Institute (APHI) experts, UNICEF Ethiopia staff, and stakeholders from district Bureau of Health (BoH), Bureau of Labour and Social Affairs (BoLSA) and Bureau of Women and Children Affairs (BoWCA), and district CBHI and PSNP coordinators.

### Data processing and analysis

We first describe the characteristics of the target population by applying the sampling weights in the descriptive analyses. Individual-level data were aggregated at the household level. All data processing and analyses were conducted using STATA software version 15.1.

To examine the impacts of CBHI enrolment on utilisation of healthcare services, we used propensity score matching (PSM) [43, 44], to account for selection into CBHI based on observable covariates, and then estimate the effect of enrolment on outpatient, maternal, and child healthcare services utilisation.

PSM allows us to construct a comparison group that comprises PSNP participating households but did not join the CBHI programme (non-treated) but with the same probability of participating in CBHI as their enrolled counterparts (treated) based on observable and controlled characteristics. The attainment of PSM’s fundamental assumptions (conditional independence assumption (CIA) or unconfoundedness, and common support) are key to reducing bias arising from observed differences between groups. Accordingly, for CIA to be met, the factors associated with CBHI enrolment among PSNP households and those factors affecting outcomes related to CBHI must be observed, i.e., the selection is solely based on observable characteristics. Further, the common support or overlap assumption also requires that households with the same characteristics (X) have a positive probability of being in both arms and have the same probability of participation between 0 and 1, such that (0 < *P*(T = 1|X) < 1) [42, 45].

We first calculate the average treatment effect (ATE), at the population level constituting differences between the treated and non-treated groups as E[Yi^T^- Yi ^C^] [46]. Next, following Smith and Todd [44] and Caliendo and Kopeinig [42] and given the above assumptions, we estimate the average treatment effect on the treated (ATT) as follows.

$${\mathrm{ATT}}_{\mathrm{PSM}} = {E}_{P(\mathrm{X}) |\mathrm{T}=1}\{E[{\mathrm{Y}}^{\mathrm{T}}|\mathrm{T }= 1, P(X)]-E[{\mathrm{Y}}^{\mathrm{C}}|\mathrm{T }= 0, P(\mathrm{X})]\}$$

where ATT is the average treatment effect on the treated for outcome Y (mean difference in outcomes between groups over the common support weighted by propensity scores), and T and C denote CBHI enrolled and non-enrolled households. *P*(X) is the probability of CBHI enrolment given the set of observable covariates *X.* Both ATE and ATT are calculated using Stata’s Treatment Effects command.

In PSM analyses, we employed a nearest neighbour algorithm with replacement (described in more detail in Additional file 1: Appendix 2). We further performed a sensitivity analysis to examine the robustness of estimates to hidden bias, described in more detail in Additional file 1: Appendix 2.

## Results

### Characteristics of sample households by CBHI enrolment status

Table 1 presents weighted descriptive statistics of covariates by CBHI enrolment status. Households enrolled with CBHI constituted 64.5% of our sample (*n* = 5,398). The average composition of households was as follows: 2.24 adults (aged 15–64 years), 1.89 children (aged 0–14 years), and 0.331 elderly aged 65 and above (≥ 65 years). The data also show that 11.9% of household heads were literate, 53.6% were currently married and 44% were females with an average age of about 53 years. We also find that 53.3% of households get water from improved sources during winter, one in every five never worried about food in the last 4 weeks but households reported to have experienced about 3 months of food insecurity in the past 12 months. Further, 17.7% and 17.5% of households have outstanding debts and experienced drought/ shortage of rainfall in the last 12 months, respectively. Households have generally high expectations about the role of CBHI in making healthcare more affordable and seeking the services easier. In addition, while households have appropriate information on how CBHI works on some key aspects, we find that only one-third and close to two-thirds know that CBHI enrolled households need to pay some costs in advance and CBHI covers medical costs related to pregnancy, respectively. Highlighting community characteristics, the study finds that the study communities are located on average 8 kms far from the nearest health centre, 24 kms from the nearest health facility with a doctor, and almost half of all the villages are located between 21 and 40 kms far from the district capital.

**Table 1:** Sample characteristics for the pooled and insurance-disaggregated households

	(1)		(2)		(3)		Mean *diff* *p*-value
	Pooled		Insured		Non-insured		
	Mean	SD	Mean	SD	Mean	SD	
Household is currently insured with CBHI^a^	0.645	0.479					
Household size by age							
Number of children [0 -14 years]	1.890	1.714	2.151	1.730	1.415	1.578	0.000***
Number of adults [15–64 years]	2.240	1.369	2.491	1.332	1.785	1.318	0.000***
Number of elders (≥ 65 years)	0.331	0.527	0.283	0.503	0.419	0.558	0.000***
Head is literate^a^	0.119	0.324	0.133	0.339	0.094	0.291	0.000***
Head is married^a^	0.536	0.499	0.620	0.486	0.385	0.487	0.000***
Head is female^a^	0.440	0.496	0.376	0.484	0.556	0.497	0.000***
Age of head (years)	52.974	14.887	51.553	13.715	55.559	16.501	0.000***
Improved water source during winter^a^	0.533	0.499	0.556	0.497	0.490	0.500	0.000***
Never worried about food last 4 weeks^a^	0.215	0.411	0.211	0.408	0.224	0.417	0.466
Number of food insecurity months last 12 months	3.205	2.738	3.193	2.620	3.228	2.940	0.762
Household has an outstanding debt^a^	0.177	0.382	0.197	0.398	0.141	0.348	0.000***
Experienced drought last 12 months^a^	0.175	0.380	0.186	0.389	0.154	0.361	0.038*
Log total annual income from PSNP	7.742	1.662	7.859	1.546	7.528	1.835	0.000***
Number of ill members last month	0.538	0.867	0.598	0.933	0.429	0.721	0.000***
Head has disability^a^	0.177	0.382	0.143	0.350	0.239	0.427	0.000***
*Perceptions about CBHI benefits*							
CBHI makes seeking health care easier^a^	0.827	0.379	0.863	0.344	0.761	0.426	0.000***
CBHI makes health care more affordable^a^	0.856	0.351	0.894	0.308	0.786	0.410	0.000***
*Understandings about CBHI:*							
CBHI covers medical costs related to pregnancy^a^	0.646	0.478	0.699	0.459	0.549	0.498	0.000***
CBHI fully covers certain drugs or surgery^a^	0.780	0.414	0.827	0.378	0.695	0.461	0.000***
No need to pay part of the cost if services covered by CBHI^a^	0.889	0.314	0.898	0.302	0.872	0.334	0.024*
Premium is not repaid even if no medical services sought^a^	0.919	0.273	0.920	0.271	0.917	0.275	0.788
Enrolled households need to pay some costs in advance^a^	0.336	0.472	0.349	0.477	0.312	0.463	0.039*
*Community and facility characteristics*							
Distance to nearest health centre (km)	7.992	10.359	8.066	10.476	7.857	10.143	0.736
Distance to nearest health facility with a doctor (km)	24.269	20.405	24.499	20.451	23.850	20.320	0.630
Nearest health facility admits people covered with CBHI^a^	0.924	0.265	0.930	0.256	0.914	0.280	0.193
Number of years the village has been in PSNP	13.232	2.950	13.247	2.961	13.205	2.931	0.811
Village distance from district capital (%)							*Chi-2*
0–10 km	19.08		18.52		20.09		
11–20 km	22.52		22.62		22.33		0.000***
21–40 km	47.92		49.51		45.01		
+ 40 km	10.49		9.35		12.57		
Observations	5398		3217		2181		

^a^Denotes dummy variables where, Yes = 1, No = 0. km denotes kilometres

^*^p < 0.05, ^**^p < 0.01, ^***^p < 0.001

Bivariate tests show that insured and non-insured households were not statistically different concerning their food insecurity experience in the last 4 weeks, the number of food insecurity months in the last 12 months, knowledge if the premium is not repaid if no medical services were sought, distance to the nearest health centre and the nearest health facility with a doctor, whether the nearest health facility admits people covered with CBHI, and the number of years the villages have been in PSNP.

Insured and Non-Insured groups were significantly different concerning household size by age, head characteristics (literacy, disability, current marital statuses, sex, and age), household profiles (sources of water during winter, having outstanding loans, shock in the past 12 months, income from PSNP, number of ill members in the past month and perceptions about CBHI benefits. Households in both arms also differ in their understandings of whether CBHI covers medical costs related to pregnancy, CBHI fully covers certain drugs or surgery, enrolled members should not pay part of the cost, if services are covered by CBHI, and if insured households need to pay some costs in advance.

### Description of outcome variables

We present descriptive statistics of outcome variables by insurance status in Table 2. Members in insured households were more likely to use outpatient healthcare services (sought care for illness last month, members sought care from health professionals, health facility visits for any medical assistance in the past 12 months, and the total number of health facility visits by all members in the past 12 months) compared to those in non-insured households.

**Table 2:** Health services utilisation characteristics by CBHI enrolment

	(1)		(2)		(3)		Mean *diff* *p*-value
	Pooled		Insured		Non-insured		
	Mean	SD	Mean	SD	Mean	SD	
**Outpatient services**							
Members sought care for illness last month^a^	0.226	0.418	0.274	0.446	0.139	0.346	0.000***
Members sought care from health professionals last month^a^	0.217	0.412	0.265	0.442	0.129	0.336	0.000***
Members consulted medical assistance from a health facility past 12 months^a^	0.476	0.499	0.559	0.497	0.324	0.468	0.000***
Number of times all members consulted medical assistance from a health facility in the past 12 months	1.777	3.059	2.237	3.426	0.940	1.988	0.000***
***N***	5398		3217		2181		
***ANC, delivery & PNC services***							
Mother got ANC from skilled provider^a^	0.495	0.500	0.515	0.500	0.439	0.497	0.020*
Four plus ANC from a skilled provider^a^	0.217	0.412	0.222	0.416	0.205	0.404	0.527
Childbirth assisted by skilled provider^a^	0.274	0.446	0.284	0.451	0.249	0.433	0.185
Childbirth delivered in a health facility^a^	0.234	0.423	0.244	0.430	0.206	0.405	0.131
PNC in health facility in the last 12 months^a^	0.332	0.471	0.356	0.479	0.267	0.443	0.001**
***N***	1564		1104		460		
**Child preventive and curative cares**							
Curative care sought for child last month^a^	0.039	0.193	0.043	0.202	0.029	0.169	0.025*
Received deworming in the last 6 months^a^	0.078	0.267	0.085	0.279	0.060	0.238	0.004**
Slept under treated bed net yesterday^a^	0.148	0.355	0.158	0.365	0.122	0.328	0.012**
Child received all vaccinations^a^	0.056	0.229	0.065	0.246	0.034	0.181	0.000***
Taken to health facility in the last 12 months^a^	0.145	0.352	0.162	0.368	0.105	0.307	0.000***
***N***	3858		2578		1280		

^a^Denotes dummy variables where, Yes = 1, No = 0

^*^p < 0.05, ^**^p < 0.01, ^***^p < 0.001

On the other hand, we find no significant differences between the two groups related to institutional delivery, if the birth was assisted by a skilled provider, and whether mother got four plus ANC from a skilled provider during the current pregnancy. However, we find that mothers of under-five children in insured households were more likely to get ANC from a skilled provider. Further, related to child(ren), those from CBHI enrolled households were more likely to be taken to a health facility for PNC in the past 12 months, have received curative care for illness last month, have received deworming in the last 6 months, slept under insecticide-treated bed net last night, have received all vaccinations, and taken to a health facility in the last 12 months for any health care services.

### Impacts of CBHI enrolment on healthcare services utilisation

We presented the detailed information on the matching algorithm utilized using online Additional file 1: Appendix 2, and predictors of CBHI enrolment using Additional file 1: Appendix 3. Information on the quality of propensity score matching including propensity score and covariate balancing are presented in online Additional file 1: Appendix 4, and sensitivity analysis and robustness check in online Additional file 1: Appendix 5.

Table 3 presents the results on the treatment effects. The average treatment effects (ATE) show that CBHI enrolment was associated with an increase in the probability of household members visiting health facilities for curative care in the last month by 8.2 percentage points. Enrolment in CBHI also leads to an increase in the probability of seeking care from a health professional in the last month by 8.4 percentage points. Looking at outpatient health services utilisation in the past 12 months, we observe that the probability that members visited a health facility to seek any medical assistance for illness or check-ups in the past 12 months increases by 13.9 percentage points and the number of health facility visits per household increases by 0.84 as a result of CBHI enrolment. There were no impacts of CBHI enrolment on antenatal care, postnatal care, skilled delivery, and child preventative or curative care services.

**Table 3:** Treatment effects of CBHI enrolment on health service utilisation

Types of healthcare services	Treatment effects			
	ATE		ATT	
	Coef	95% CI	Coef	95% CI
***Outpatient health services (N* = *5,386)***				
Sought care for illness last month	0.082^***^	[0.053,0.111]	0.091^***^	[0.055,0.127]
Sought care from health professionals last month	0.084^***^	[0.055,0.113]	0.091^***^	[0.056,0.127]
Visited health facility in the last 12 months	0.139^***^	[0.105,0.174]	0.138^***^	[0.097,0.179]
Number of total health facility visits in the last 12 months	0.840^***^	[0.640,1.040]	0.928^***^	[0.674,1.183]
***ANC, delivery & PNC services (N* = *1,564)***				
Got ANC from a skilled provider	0.017	[-0.053,0.087]	0.005	[-0.075,0.086]
Received 4 + ANC visits	0.004	[-0.056,0.063]	0.002	[-0.067,0.071]
Birth assisted by a skilled professional	-0.008	[-0.072,0.056]	-0.022	[-0.096,0.052]
Delivery in a health facility	-0.005	[-0.065,0.056]	-0.019	[-0.090,0.052]
Had PNC visit last 12 months	0.029	[-0.040,0.097]	0.015	[-0.064,0.095]
***Child preventive & curative services (N* = *3,850)***				
Got curative care last month	-0.009	[-0.026,0.009]	-0.011	[-0.033,0.010]
Received deworming in the last 6 months	-0.005	[-0.027,0.018]	-0.010	[-0.037,0.017]
Slept under insecticide-treaded bed net yesterday	0.005	[-0.025,0.035]	0.004	[-0.032,0.040]
Received all vaccinations	0.015	[-0.004,0.033]	0.016	[-0.006,0.034]
Visited health centre in the last 12 months	0.008	[-0.022,0.038]	0.002	[-0.034,0.038]

95% confidence intervals in brackets; Std. Err. adjusted for clustering in villages

^*^p < 0.05, ^**^p < 0.01, ^***^p < 0.001

The average treatment effects on the treated (ATT) estimates further strengthen the findings from ATE estimates, indicating CBHI has resulted in more utilisation of outpatient healthcare services among insured households. As expected, ATT estimates are larger than ATE estimates, except for the health facility visits in the past 12 months where the ATT is slightly smaller than the ATE. Among insured households, CBHI enrolment increased the likelihood of seeking healthcare services for illness in the previous month by 9.1 percentage points, seeking healthcare from professional care provider by 9.1 percentage points, and the probability of members visiting health facilities over the past 12 months by 13.8 percentage points. Furthermore, regarding the intensity of healthcare visits, we find that CBHI enrolment among insured households increased the mean number of health facility visits per household by 0.93. Also, we find no significant impacts of CBHI participation on ANC, institutional delivery, PNC, and child preventive and curative health services utilisation among treated households (Table 3).

In our sensitivity analyses (Additional file 1: Appendix 5), we do not find that CBHI enrolment is determined by unobservable characteristics. Moreover, in a robustness check of our PSM findings using a more restricted calliper width and increasing the number of untreated subjects to be matched, we show that the findings described above are robust (Additional file 1: Appendix 5).

## Discussion

### Statement of principal findings

Household enrolment in CBHI was 64.5%. The study finds that enrolment in CBHI increases the likelihoods of visiting health facilities for curative care in the past one month by 8.2 percentage points, seeking care from a health professional by 8.4 percentage points, visiting a health facility to seek any medical assistance for illness and check-ups in the past 12 months by 13.9 percentage points, and the number of health facility visits per household by 0.84. However, we didn’t find statistically significant impacts of CBHI enrolment on antenatal care, skilled delivery, postnatal care, and child preventative curative services.

### Strengths and weaknesses of the study

The study adds insights into the effects of CBHI enrolment on health services utilisation patterns among extremely poor and most vulnerable households targeted by Ethiopia’s cash transfer programme (the PSNP). In addition, the study also provides early and cross-sectional evidence on the potential effects of integrating the two largest social protection programmes on health service utilisations among extremely poor households.

The study has some weaknesses worth mentioning. We used cross-sectional data generated from one region only in the country (out of ten regions and two city administrations) and exclusively PSNP-participating households. Thus, findings cannot be generalized to the whole country which also includes non-PSNP and well-to-do households. In addition, estimates may be biased by self-selection into voluntary CBHI enrolment based on unobservable characteristics and from omitted variables. We also lack information on the temporal ordering of CBHI enrolment and the outcome variables. This could have been addressed well using longitudinal data. Further, the study assumes that CBHI benefit packages and refunding requirements and procedures, which may influence enrolment decisions and service utilisations, are similar across districts.

### Comparison with other studies and possible explanations

Our findings that CBHI enrolment led to greater outpatient services use is supported by previous studies in Ethiopia on the general population, including Shigute et al*.* [30], Mebratie et al*.* [24], Mebratie et al*.* [25], and Tilahun et al*.* [29]. Shigute et al*.* [30] analysed the effects of CBHI alone and combined with PSNP on the use of modern healthcare utilisation for outpatient care and modern health facility visits in Ethiopia using three rounds of individual-level panel data. In line with our study findings, they also reported that CBHI nudges the probability of using modern healthcare services (visiting a modern health care facility for outpatient care services) by 2.3 percentage points and the number of visits to modern health facilities by 0.07 among adult members in the pooled sample. For the PSNP-only sub-sample, they find that CBHI increases utilisation of modern outpatient healthcare services by 4 percentage points. Although this is larger compared to the pooled sample among adult members, both estimates are by far smaller than the impacts in our study. In contrast to our study, they find no significant impact of CBHI enrolment on the number of modern health facility visits among the PSNP-participating sub-sample. Another study by Mebratie et al*.* [25] also showed that enrolment in pilot CBHI scheme increased utilisation of outpatient healthcare services at public health facilities by 30–41 percentage points and increases the number of public health facility visits by 0.05–0.07 in the past 2 months.

Some important differences between our and the two studies may have resulted in the variations in the estimated impacts. First, the two existing studies used a more general population (among which a sub-sample of households were participating in PSNP) while our sample is comprised entirely from PSNP-participating households. Second, while data used for both previous studies came from the CBHI pilot phase, our data came from a recent large-scale baseline survey conducted in rural Amhara, after CBHI had been scaled up nationally. Thus, our findings have greater generalizability concerning CBHI impacts among vulnerable groups targeted by the PSNP. Third, related to the recall periods, we used the previous one month to ask about the use of outpatient services by any of the household members for illness, but the 12 months period to ask the number of total health facility visits by all household members for outpatient care. They used the past two months for a recall which may fail to capture some of the health facility visits made in the year compared to the 12-month period. But, their approach is accompanied by less recall biases. However, both studies used panel data while our study relied on cross-sectional data. Tilahun et al*.* [29], using cross-sectional data from one district in Amhara region, also find that membership in mutual health insurance increases the likelihood of using healthcare by 25.2 percentage points. However, it is not clear if the study households were also participating in PSNP.

Our null findings related to maternal and child healthcare services utilisation and health insurance enrolment are consistent with other studies from different settings, including having received four or more ANC visits in Rwanda [27]. They also find no impact of health insurance on receiving at least four ANC services in Rwanda. On the other hand, Fernandes et al*.* [47] find that insured women were less likely to use skilled birth attendance during delivery in Jordan. However, our results contrast with maternal and child health-related findings reported elsewhere. Health insurance increases the likelihood of receiving at least four ANC visits in Jordan [47], Ghana and Indonesia [27], increased the probability of health facility-based delivery in Ghana, Rwanda and Indonesia [27], Tanzania [48], and Egypt [49]. In Ethiopia, Atnafu and Gebremedhin [31] find that the CBHI programme has a positive effect on the use of curative healthcare services for children in households with at least one child experienced illness in the past 4 weeks. We posit that the non-significant effects of CBHI enrolment on the maternal and child healthcare services could be due to the free provision of such services in the country at public health facilities. These facilities are also the sole health service providers for CBHI insured households. This means that both insured and non-insured households have equal access to all maternal and child healthcare services at public health facilities. In addition to the free availability of several maternal and child healthcare services at the health posts, information and sensitization efforts such as the behavioural change communications (BCCs) sessions targeting all PSNP households may have resulted in better awareness and knowledge about the importance and availability of maternal and child preventive and curative services in nearby health posts among insured and non-insured households alike. Health posts are the first point of contact public health facilities for rural households in rural villages.

### Possible mechanisms

The study also provides explanations of two potential causal pathways between enrolment in CBHI and outpatient health services utilisation. With no appropriate financial mechanisms, healthcare seeking in poorly functioning health systems is associated with a risk of catastrophic expenditures [50]. Borde et al. [51] find that in Ethiopia the average direct out-of-pocket healthcare expenditures were USD 32 per month, the average indirect out-of-pocket healthcare expenditures were USD 15 per month and the average catastrophic healthcare expenditure at 10% of threshold was 40%. Accordingly, consistent with past related studies [52–55], our first hypothesised pathway is that CBHI may have reduced the high out-of-pocket health spending, thereby encouraging utilisation of healthcare services among the PSNP-participating households. In this regard, an evaluation of the pilot CBHI in Ethiopia also finds that 37% of CBHI members joined the programme to primarily reduce out-of-pocket expenditure when seeking health care, and 35% joined CBHI to seek healthcare more frequently [56].

We also hypothesised that CBHI’s role to empower women could be another pathway linking CBHI enrolment and enhanced utilisation of outpatient healthcare services. In Ethiopia, men are considered to be the primary breadwinners and have the decision-making power in all household financial matters including spending on healthcare. This means that in uninsured households and whenever service seekers have to pay service fees upfront, some members, including women, may not be able to get or delay getting the treatment due to a lack of financial autonomy. In this regard, earlier evidence showed that CBHI empowered women – enabled them to seek essential health care whenever needed without requesting money and permission from male heads of household [57]. In support of this evidence, a recent study by Messner et al. [58] also finds that women in CBHI insured households are more likely to seek treatment for themselves or their children without financial support from a male head. For example, one of the study respondents in Messner et al*.* [58] study stated that:

“Unfortunately majority of us, women, don’t have income of our own. We rely on our husband’s money in order to pay for the medical bill. But if we have this card, we don’t have to ask our husbands for money whenever we are sick. In addition, our husbands may not be at home when we get sick. Hence, having this card will allow us to go to the health centre without waiting on our husbands.” — Married woman, age 24–45, Tigray.

While there could be more mechanisms, more rigorous studies are needed to fully understand the causal mechanisms between CBHI enrolment and improved outpatient health service utilisations.

### Policy implications

The government of Ethiopia implemented several policy measures to enhance households' protection against financial risks in accessing essential health services and to improve health service utilisation. Community-based health insurance is one of these measures. The current health sector transformation plan aims to accelerate the progress towards full coverage of essential health services and protecting people from financial hardship, including those in currently underserved populations [59]. Achieving UHC entails the achievement of all components of UHC (availability of all essential health services at each service delivery with an acceptable level of quality, effective coverage of essential health services, and ensuring financial risk protection) to all population subgroups. Our findings suggest that enrolment in CBHI is one of the promising strategies towards UHC and plays a vital role to help vulnerable and PSNP-participating households to access some of the available essential outpatient services. However, further evidence is still needed in other dimensions of UHC such as on the availability of all essential health services at all public health facilities, mainly at primary health facilities, quality of care, and individual level coverage. Moreover, since enrolment into Ethiopia’s CBHI is voluntary, the poorest non-PSNP households may still be excluded from the programme. A recent study in one of the study districts also finds that extremely poor and most vulnerable households to extreme poverty who are not receiving conditional cash transfers are less likely to join CBHI [60]. To place CBHI better as a tool towards UHC, the government may implement more measures such as universal eligibility for insurance with a substantial premium subsidy and the universal individual-level exemption for some vulnerable groups such as pregnant women from paying premiums [61].

Our study also provides some evidence on the role of CBHI in ensuring equity in healthcare in the informal sector. Related to this, although equity should be looked at both at the CBHI enrolment and service utilisation stages [62], improved utilisation of outpatient healthcare services by the ensured vulnerable households suggests that CBHI also contributes partly towards equity in healthcare among some of the most vulnerable groups in the country. Past studies suggest that enrolment among the poor and marginalized households can be also enhanced through improving roads and public transport systems [62] and premium subsidy and fee waiver [63, 64].

Given that the per capita health facility visits in Ethiopia (0.9 visits annually in 2019) has so far been far below the World Health Organization (WHO) recommended level—2.5 visits per capita per year [65], the significant effect of CBHI enrolment on health facility visits indicates the programme has a promising potential for Ethiopia to reach the WHO recommended level of per capita health facility visit. More importantly, the significant impact of CBHI enrolment on health facility visits among the most vulnerable households underscores the critical role of CBHI to ensure health equity in the country and to leave no one behind.

### Future research areas

Existing literature shows that health insurance programmes, including CBHI, are prone to moral hazard problems which occur when enrolment in health insurance is followed by increases in healthcare consumption and a reduction in preventive measures [66–69]. However, as this is not always the case due to preferred and needed healthcares [70], future studies may investigate whether this problem exists among insured or not in Ethiopia’s CBHI programme. Past studies in other related settings argued that due to low availability and utilisations of healthcare services and high unmet demand, improvements in health services utilisations among such populations may not be due to moral hazard, but the impact of health insurance [21]. Future studies may also explore if an adverse selection problem exists in the CBHI enrolment which in turn could affect the health service utilisation decisions.

Access to information about CBHI benefit packages (entitlements) and available health services at different health facilities could also influence CBHI enrolment decisions as well as health services utilisations. Future studies may investigate how exposure to various information and awareness sessions such as the behavioural change communications (BCCs) and information campaigns affect health services utilisation by PSNP-participating households. Other potential areas of investigation include how perceived or actual institutional arrangements such as refunding and referral systems between health facilities affect health service utilisations. Evidence on the individual level health service utilisation among insured households using CBHI card can also give highlights about the intra-household gender and power dynamics in using specific health services using CBHI card. Finally, future studies may also explore the pro-poorness of the CBHI among PSNP-participating households in terms of their health outcomes.

## Conclusions

This study evaluated the impacts of enrolment in CBHI on the utilisation of outpatient, maternal, and child preventive and curative healthcare services among the most vulnerable rural households in Ethiopia. Approximately two-thirds of the sample households are insured in CBHI. We find that enrolment in CBHI was positively associated with using more outpatient healthcare services including visiting health facilities for curative care in the past one month, seeking care from a health professional, visiting a health facility to seek any medical assistance for illness and check-ups in the past 12 months, and the number of health facility visits per household. However, the study finds no significant impacts of membership in CBHI on maternal and child healthcare services. The study provides insights on the role of CBHI among safety net programme beneficiaries to achieve UHC and health equity and increase the per capital annual health facility visits. The evidence can contribute to policy making aimed to integrate the two largest social protection programmes (CBHI and PSNP) in the country and mitigate the adverse impacts of multidimensional poverty.

## Supplementary Information

**Additional file 1: Appendix 1.** Impact evaluation design details. **Appendix 2.** Methods: Details on Matching Algorithm and Sensitivity Analysis . **Appendix 3.** Predictors of enrolment into CBHI. **Appendix 4.** Propensity Score Matching (PSM) Results. **Appendix 5.** Sensitivity analysis and robustness check. **Appendix 6.** Variable types and measurements [71–79].

## Data Availability

The datasets generated and/or analysed during the current study are not publicly available since the data is part of an ongoing study which is not yet completed. It is expected to be made available no sooner than one year after the final impact evaluation report is published, pending UNICEF and Government approval and can be available from the corresponding author on reasonable request.

## Abbreviations

APHI: Amhara Public Health Institute
ANC: Antenatal Care
ARI: Acute Respiratory Illness
ATE: Average Treatment Effect
ATT: Average Treatment Effect on the Treated
BCC: Behavioural Change Communications
BoH: Bureau of Health
BoLSA: Bureau of Labour and Social Affairs
BoWCA: Bureau of Women and Children Affairs
CBHI: Community-Based Health Insurance
CIA: Conditional Independence Assumption
DHS: Demographic and Health Survey
DTP: Diphtheria, Tetanus and Pertussis
ECO: Ethiopia Country Office
ETB: Ethiopian Birr
HSTP: Health Sector Transformation Plan
HSDP: Health Sector Development Programme
ISNP: Integrated Safety Net Programme
ITBN: Insecticide-Treated Bednet
LMIC: Lower- and Middle-Income Countries
MICS: Multiple Indicator Cluster Survey
MoARD: Ministry of Agriculture and Rural Development
MoLSA: Ministry of Labour and Social Affairs
NSPP: National Social Protection Policy
NSPS: National Social Protection Strategy
ORT: Oral Rehydration Therapy
PDS: Permanent Direct Support
PHC: Primary Health Care
PNC: Post-natal Care
PSM: Propensity Score Matching
PSNP: Productive Safety Net Programme
PW: Public Works
SDGs: Sustainable Development Goals
SPAP: Social Protection Action Plan
UHC: Universal Health Coverage
UN: United Nations
UNICEF: United Nations Children’s Fund
USD: United States Dollars
WHO: World Health Organization
