# Supplementary material for: Impact of community-based health insurance on health services utilisation among vulnerable households in Amhara region, Ethiopia
Source: BMC Health Serv Res. 2023 Jan 19;23:55. doi: 10.1186/s12913-023-09024-3 (PMC9850585; doi:10.1186/s12913-023-09024-3)
Supplement: Supplementary file 1 — Additional file 1: Appendix 1. Impact evaluation design details. Appendix 2. Methods: Details on Matching Algorithm and Sensitivity Analysis . Appendix 3. Predictors of enrolment into CBHI. Appendix 4. Propensity Score Matching (PSM) Results. Appendix 5. Sensitivity analysis and robustness check. Appendix 6. Variable types and measurements [71–79]. [file 12913_2023_9024_MOESM1_ESM.docx]

**APPENDIX**

**List of Appendices:**

Appendix 1. Impact evaluation design details

Appendix 2. Methods: Details on Matching Algorithm and Sensitivity Analysis

Appendix 3. Predictors of enrolment into CBHI

Appendix 4. Propensity Score Matching (PSM) Results

Appendix 5. Sensitivity analysis and robustness check

Appendix 6. Variable types and measurements

**Appendix 1. Impact evaluation design details**

The pilot ISNP impact evaluation aims to integrate the Ethiopian Productive Safety Net Program (PSNP), rolled out in 2005, and a Community-Based Health Insurance (CBHI), piloted in 2011 and scaled up substantially in subsequent years. A mixed-method impact evaluation is being conducted by UNICEF Office of Research – Innocenti, UNICEF – Ethiopia Country Office (UNICEF ECO), University at Buffalo, and a local research partner, Frontier*i*, formerly called BDS – Centre for Development Research. The evaluation is being carried out in 4 rural woredas (districts) of Amhara region, namely Libo Kemkem and Dewa Chefa as treatment districts (households receiving PSNP plus integrated PSNP/CBHI implementation activities including facilitation to CBHI enrolment, nutrition information through Behavioural Change Communication (BCC) sessions, and case management through social workers and community care coalition support) and Ebinat and Artuma Fursi as comparison districts (households receiving the PSNP but not the integrated plus components). The treatment districts were selected purposively based on the availabilities of CBHI in the district, UNICEF Ethiopia country office (ECO) nutrition interventions, linkages to other UNICEF interventions, and district accessibility and practicality for UNICEF ECO support. The comparison districts were selected based on their similarities with treatment districts in socio-demographic, health service supply, program organization, culture/ ethnicity, and ecological characteristics. Thus, the treatment and their respective comparison districts are geographically close and similar culturally and economically. The comparison districts were selected from the same zones where the treatment districts were located — Libo Kemkem and Ebinat districts from Debub/South Gondar Zone, and Artuma Fursi and Dawa Chefa from Oromo Zone.

To determine the minimum sample size for the impact evaluation, power calculations were conducted based on expected means and impacts on the indicators including individuals using health services during the last month (among those who were sick), consulting a health practitioner or traditional healer or visited a health facility in the last 4 months, enrolment in CBHI, children aged 6-23 months who receive a minimum acceptable diet, children aged 12-23 months with all basic vaccinations, children aged 12-23 months with no vaccination, children 12-23 months with age-appropriate vaccinations and antenatal care for last pregnancy during last year from a skilled provider.

Baseline quantitative and qualitative interviews were implemented between December 2018 and February 2019. Then a qualitative follow-up was implemented in January and February 2019. Mixed method endline interviews are expected to be implemented in late 2022.

**Appendix 2. Methods: Details on Matching Algorithm and Sensitivity Analysis**

**Matching Algorithm**

We used the nearest neighbour algorithm that identifies the closest households in the comparison arm that best matched households in the treatment arm based on the propensity scores. This algorithm also helps to differentiate between households in the two arms which guarantees that all insured households are successfully matched, thereby providing the most information on the two groups (71). Although the matching can be done on one-to-one or one-to-many (often the number of untreated subjects goes up to five to be matched per a treated subject), Austin (72) recommends matching either 1 or 2 untreated subjects to each treated subject. Accordingly, since we have 3,217 insured and 2,181 non-insured households (1:1.475 ratio between non-treated and treated households), we implemented a one-to-one nearest neighbour approach with replacement. We also used a calliper width of 0.2 standard deviations of a logit of propensity scores (this is 0.04 in our case)^[[1]](#footnote-1)^ which has a superior performance (73,74), so that the nearest neighbour matching method uses one comparison household available within the calliper.

**Sensitivity analysis**

Since only observed differences between treatment and comparison groups can be controlled through adjusting covariates and propensity scores, unobserved differences may still exist between groups which may result in hidden bias due to their effects on assignment into treatment and the outcomes of interest (42,75). In such cases, it is recommended to run a sensitivity analysis to examine the robustness of estimates to such hidden bias. We followed Becker and Caliendo (75) to compute the Mantel-Haenszel bounds (mhbounds) to check for sensitivity estimated average treatment effects for binary outcomes and Rosenbaum bound (rbounds) for continuous outcomes. It must be noted that the approach does not measure whether there are unobserved variables that influence selection into treatment — to test for the unconfoundedness assumption, instead it indicates the extent to which significant results were dependent on this untestable assumption. As such, if the significant results were sensitive to these unobserved variables, one should consider using alternative estimation strategies (42,75).

**Appendix 3. Predictors of enrolment into CBHI**

Table A1 presents weighted binary logistic regression results on the predictors of enrolment in CBHI and reports odds ratios in three models. Standard errors were adjusted for clustering at the kebele (village) level. Model 1 controls for demographic and socioeconomic status characteristics. Then, we included CBHI perceptions and understanding indices in Model 2. In Model 3, we further added community and health facility characteristics. In all the estimations, district fixed effects are included.

We find that the following characteristics were significantly and positively associated with CBHI enrolment: the number of children and adult members, married head of households, female head, increasing age of the head, households who have access to improved water sources during winter season, wealth status, income from PSNP in the last 12 months, and the number of ill members last month. Accordingly, in Model 1, the results show that the odds of CBHI enrolment among PSNP households increases for increasing in the number of children aged 0-14 years (OR=1.090; CI:1.032–1.152), adults aged 15-64 years (OR=1.211; CI:1.126–1.303), among married heads (OR=1.739; CI: 1.348–2.243) and households headed by females (OR=1.386; CI:1.093–1.759). It was also found that the odds of households’ enrolment in CBHI increases as the age of the household head increases (OR=1.045; CI:1.014–1.077). Further, CBHI enrolment was positively associated with the number of household members who reported illness last month (OR=1.186; CI: 1.083–1.300). Households with increased wealth status and higher income from PSNP in the previous 12 months were also more likely to enrol in CBHI. Characteristics not associated with CBHI enrolment in our sample included the number of elders in the household, heads’ literacy status, experiences of any shocks in the previous 12 months, the number of ill household members last month, and whether the household head has a disability or not.

In Model 2, while the variables discussed above remained consistent and significantly associated with CBHI enrolment, we also find that CBHI enrolment is positively associated with a good understanding of how CBHI works and its benefit packages (OR=1.714; CI: 1.437–2.044 1.451–2.070) and if the respondent thinks that CBHI makes seeking health care easier and more affordable (OR=1.231; CI: 1.134–1.336). We find no significant association between CBHI enrolment decision and health facility and community characteristics (Model 3).

Table A1: Logistic regression on predictors of enrolment in CBHI (Dependent variable: Household currently covered by CBHI; Odds Ratios reported)

|  | (1) | | (2) | | (3) | |
| --- | --- | --- | --- | --- | --- | --- |
|  | Model 1 | | Model 2 | | Model 3 | |
| Household size by age |  |  |  |  |  |  |
| Number of children [0 -14 years] | 1.090^**^ | [1.032,1.152] | 1.098^**^ | [1.038,1.161] | 1.098^**^ | [1.037,1.162] |
| Number of adults [15-64 years] | 1.211^***^ | [1.126,1.303] | 1.223^***^ | [1.137,1.316] | 1.221^***^ | [1.135,1.313] |
| Number of elders (≥65 years) | 0.979 | [0.827,1.158] | 0.974 | [0.821,1.156] | 0.966 | [0.814,1.146] |
| Head is literate | 0.919 | [0.717,1.180] | 0.883 | [0.687,1.134] | 0.877 | [0.681,1.128] |
| Head is married | 1.739^***^ | [1.348,2.243] | 1.749^***^ | [1.340,2.282] | 1.724^***^ | [1.318,2.254] |
| Head is female | 1.386^**^ | [1.093,1.759] | 1.377^*^ | [1.073,1.768] | 1.348^*^ | [1.047,1.735] |
| Age of head | 1.045^**^ | [1.014,1.077] | 1.044^**^ | [1.012,1.077] | 1.044^**^ | [1.012,1.077] |
| Head's age squared | 1.000^**^ | [0.999,1.000] | 1.000^**^ | [0.999,1.000] | 1.000^**^ | [0.999,1.000] |
| Household has improved water source during winter | 1.496^***^ | [1.274,1.757] | 1.446^***^ | [1.235,1.692] | 1.428^***^ | [1.222,1.669] |
| Household never worried about food last 4 weeks | 0.789^*^ | [0.636,0.980] | 0.801^*^ | [0.643,0.998] | 0.797^*^ | [0.642,0.989] |
| Number of food insecurity months | 1.011 | [0.982,1.041] | 1.011 | [0.981,1.042] | 1.009 | [0.980,1.040] |
| Household has an outstanding debt | 0.890 | [0.735,1.080] | 0.843 | [0.691,1.029] | 0.851 | [0.697,1.040] |
| Household experienced drought/ irregular rain | 0.941 | [0.779,1.137] | 0.899 | [0.746,1.083] | 0.921 | [0.761,1.113] |
| Log total annual income from PSNP | 1.071^**^ | [1.023,1.122] | 1.063^**^ | [1.015,1.114] | 1.061^*^ | [1.014,1.110] |
| Wealth index |  |  |  |  |  |  |
| 2^nd^ quartiles | 1.212^*^ | [1.013,1.450] | 1.225^*^ | [1.017,1.475] | 1.232^*^ | [1.029,1.475] |
| 3^rd^ quartiles | 1.977^***^ | [1.599,2.444] | 2.011^***^ | [1.641,2.466] | 2.027^***^ | [1.652,2.488] |
| 4^th^ quartiles | 2.438^***^ | [1.898,3.133] | 2.378^***^ | [1.855,3.047] | 2.418^***^ | [1.878,3.114] |
| Number of ill members last month | 1.186^***^ | [1.083,1.300] | 1.164^**^ | [1.062,1.276] | 1.158^**^ | [1.059,1.268] |
| Head has disability | 0.864 | [0.722,1.033] | 0.926 | [0.770,1.114] | 0.926 | [0.770,1.113] |
| CBHI understandings Index |  |  | 1.714^***^ | [1.437,2.044] | 1.674^***^ | [1.390,2.015] |
| CBHI perceptions Index |  |  | 1.231^***^ | [1.134,1.336] | 1.247^***^ | [1.150,1.352] |
| Village distance from the district capital |  |  |  |  |  |  |
| 11-20 km |  |  |  |  | 1.166 | [0.852,1.596] |
| 21-40 km |  |  |  |  | 1.132 | [0.867,1.479] |
| +40 km |  |  |  |  | 0.698 | [0.480,1.016] |
| Number of years the village has been in PSNP |  |  |  |  | 0.987 | [0.966,1.008] |
| Distance to the nearest health centre |  |  |  |  | 1.002 | [0.991,1.013] |
| Distance to the nearest health facility with a doctor |  |  |  |  | 0.999 | [0.993,1.005] |
| Nearest health facility admits people covered with CBHI |  |  |  |  | 0.861 | [0.637,1.163] |
| District Fixed Effects | Yes |  | Yes |  | Yes |  |
| Observations | 5398 |  | 5398 |  | 5398 |  |

Exponentiated coefficients; 95% confidence intervals in brackets; Std. Err. adjusted for clustering in village; ^*^*p*< 0.05, ^**^*p*< 0.01, ^***^*p*< 0.001

**Appendix 4. Propensity Score Matching (PSM) Results – the quality of matching**

Propensity scores were calculated with a logistic regression model that had 24 covariates (Model 3, Table A1). As balance checks, we presented propensity score balancing according to healthcare services categories (different sample sizes) and covariate balances.

**Propensity Score Balancing: Density graphs**

We present the distribution of propensity scores using kernel density (Figure A1). Visual inspections of the propensity score distributions between treated (insured) and non-treated (non-insured) groups suggest that the densities of the propensity scores are more similar after matching for all outcomes considered in the study. The propensity score distributions show clear overlapping between insured and non-insured groups post matching. After matching, we remained with 5,386 households (12 non-insured households violated the overlap assumption) for outpatient services; 1,562 households (2 non-insured households violated the overlap assumption) for maternal services, and 3,850 households (8 non-insured households violated the overlap assumption) for child preventive and curative services. Households who violated the overlap assumption were dropped from the treatment effects analyses.


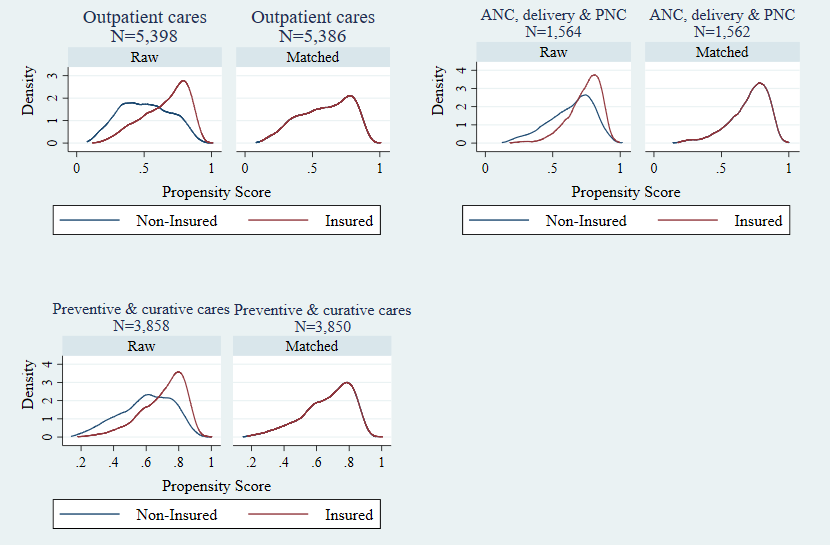


**Figure A1: Density graphs for propensity scores before and after matching**

**Covariate Balancing**

In addition to the density graphs for propensity scores, we also checked the covariate-specific balances and used the recommended absolute standardized difference in means or proportions (ASMD) method (76). This method is more robust in terms of sample size and covariate distribution requirements compared to other balance diagnostics and helps to identify variables that did not achieve balance and, hence, modify the propensity scores to ensure balance of covariates such as by adding higher-order variables (77). For each covariate, standardized mean differences (SMD) were calculated before (raw) and after (matched) implementing PSM. Covariate balance was assessed by the number of covariates with SMD, 0.1 (< 10% for the ASMD is considered good matching), which is commonly used in the medical literature (77,78). Additionally, we used the ratio of variances of treatment and comparison groups for further balance checking. If variance ratios are 1.0, balance is considered good and acceptable while a value below 2.0 is generally acceptable (77,79). According to Rubin (79), variance ratios below 0.5 and above 2 are outside the acceptable range to ensure covariate balance between the two groups (79). Summary of covariate balance before and after matching presented using Tables A2-A4 show that standardized mean and proportion differences for matched covariates are below 0.1 for all covariates for the three analyses and the variance ratios are mostly close to 1.0. The values range between 0.782 for CBHI understandings Index and 1.103 for the number of years the village has been in PSNP for the outpatient services, between 0.740 related to distance to the nearest health centre and 1.338 related to the number of years the village has been in PSNP for maternal healthcare utilization and between 0.823 related to CBHI understandings Index and 1.156 for distance to the nearest health facility with a doctor for child preventive and curative healthcare services utilization. These show that we have achieved covariate balance between insured and non-insured groups on observed and controlled characteristics for the three analyses, suggesting that both groups are comparable on these observables.

Table A2: Covariate balance summary for outpatient services utilization (N=5,386)

| Covariates | Standardized differences | | Variance ratio | |
| --- | --- | --- | --- | --- |
|  | Raw | Matched | Raw | Matched |
| Household size by age |  |  |  |  |
| Number of children [0 -14 years] | 0.457 | -0.003 | 1.329 | 0.967 |
| Number of adults [15-64 years] | 0.555 | 0.002 | 1.113 | 0.993 |
| Number of elders (≥65 years) | -0.258 | 0.022 | 0.904 | 1.070 |
| Head is literate | 0.136 | 0.006 | 1.402 | 1.015 |
| Head is married | 0.479 | 0.036 | 1.127 | 1.006 |
| Head is female | -0.364 | -0.038 | 1.025 | 1.000 |
| Age of head | -0.319 | 0.016 | 0.750 | 1.047 |
| Head's age squared | -0.354 | 0.022 | 0.704 | 1.070 |
| Household has improved water source during winter | 0.166 | 0.016 | 0.974 | 0.997 |
| Household never worried about food last 4 weeks | 0.016 | -0.049 | 1.022 | 0.935 |
| Number of food insecurity months | -0.047 | 0.006 | 0.785 | 0.921 |
| Household has an outstanding debt | 0.152 | -0.035 | 1.337 | 0.939 |
| Household experienced drought/ irregular rain | 0.074 | -0.005 | 1.142 | 0.992 |
| Log total annual income from PSNP | 0.174 | 0.055 | 0.781 | 0.934 |
| Wealth index |  |  |  |  |
| 2^nd^ quartiles | -0.166 | 0.039 | 0.831 | 1.044 |
| 3^rd^ quartiles | 0.219 | 0.016 | 1.306 | 1.019 |
| 4^th^ quartiles | 0.434 | -0.012 | 1.779 | 0.987 |
| Number of ill members last month | 0.208 | 0.011 | 1.689 | 1.039 |
| Head has disability | -0.240 | 0.020 | 0.751 | 1.024 |
| CBHI understandings Index | 0.284 | -0.038 | 0.853 | 0.782 |
| CBHI perceptions Index | 0.272 | -0.010 | 0.593 | 0.994 |
| Village distance from the district capital |  |  |  |  |
| 11-20 km | 0.023 | -0.019 | 1.026 | 0.979 |
| 21-40 km | 0.070 | 0.003 | 1.014 | 1.001 |
| +40 km | -0.115 | 0.007 | 0.726 | 1.022 |
| Number of years the village has been in PSNP | 0.026 | -0.008 | 0.966 | 1.103 |
| Distance to the nearest health centre | 0.004 | 0.015 | 1.026 | 1.069 |
| Distance to the nearest health facility with a doctor | 0.001 | 0.011 | 0.915 | 1.017 |
| Nearest health facility admits people covered with CBHI | 0.051 | 0.013 | 0.862 | 0.962 |

Table A3: Covariate balance summary for ANC, health facility delivery and PNC services utilization (N=1,562)

| Covariates | Standardized differences | | Variance ratio | | |
| --- | --- | --- | --- | --- | --- |
|  | Raw | Matched | Raw | Matched | |
| Household size by age |  |  |  |  | |
| Number of children [0 -14 years] | 0.276 | -0.007 | 1.082 | 1.066 | |
| Number of adults [15-64 years] | 0.225 | -0.045 | 1.269 | 1.064 | |
| Number of elders (≥65 years) | -0.002 | -0.030 | 0.953 | 0.919 | |
| Head is literate | 0.102 | 0.094 | 1.185 | 1.178 | |
| Head is married | 0.244 | -0.044 | 0.724 | 1.067 | |
| Head is female | -0.228 | 0.023 | 0.748 | 1.033 | |
| Age of head | 0.031 | -0.025 | 0.808 | 0.975 | |
| Head's age squared | 0.001 | -0.024 | 0.780 | 0.883 | |
| Household has improved water source during winter | 0.137 | 0.026 | 0.981 | 0.996 | |
| Household never worried about food last 4 weeks | -0.004 | -0.027 | 0.993 | 0.964 | |
| Number of food insecurity months | 0.024 | -0.031 | 0.902 | 1.005 | |
| Household has an outstanding debt | 0.031 | 0.006 | 1.040 | 1.008 | |
| Household experienced drought/ irregular rain | -0.012 | -0.025 | 0.979 | 0.957 | |
| Log total annual income from PSNP | 0.189 | 0.043 | 0.562 | 0.879 | |
| Wealth index |  |  |  |  |  |
| 2^nd^ quartiles | -0.217 | -0.010 | 0.743 | 0.985 |  |
| 3^rd^ quartiles | 0.040 | -0.022 | 1.027 | 0.986 |  |
| 4^th^ quartiles | 0.254 | 0.034 | 1.146 | 1.016 |  |
| Number of ill members last month | 0.201 | -0.017 | 1.516 | 0.955 |  |
| Head has disability | -0.066 | 0.059 | 0.839 | 1.182 |  |
| CBHI understandings Index | 0.289 | -0.007 | 0.689 | 0.828 |  |
| CBHI perceptions Index | 0.365 | -0.000 | 0.502 | 0.978 |  |
| Village distance from the district capital |  |  |  |  |  |
| 11-20 km | -0.076 | -0.026 | 0.921 | 0.972 |  |
| 21-40 km | 0.114 | 0.076 | 1.022 | 1.018 |  |
| +40 km | -0.114 | -0.051 | 0.722 | 0.872 |  |
| Number of years the village has been in PSNP | 0.030 | -0.057 | 0.938 | 1.338 |  |
| Distance to the nearest health centre | 0.051 | -0.099 | 1.140 | 0.740 |  |
| Distance to the nearest health facility with a doctor | 0.022 | -0.006 | 1.213 | 1.217 |  |
| Nearest health facility admits people covered with CBHI | 0.109 | 0.015 | 0.690 | 0.951 |  |

Table A4: Covariate balance summary for child-related preventive and curative healthcare services utilization (N=3,850)

| Covariates | Standardized differences | | Variance ratio | |
| --- | --- | --- | --- | --- |
|  | Raw | Matched | Raw | Matched |
| Household size by age |  |  |  |  |
| Number of children [0 -14 years] | 0.245 | 0.041 | 1.225 | 1.086 |
| Number of adults [15-64 years] | 0.386 | 0.010 | 1.022 | 1.003 |
| Number of elders (≥65 years) | -0.161 | -0.029 | 0.860 | 0.969 |
| Head is literate | 0.119 | 0.029 | 1.299 | 1.067 |
| Head is married | 0.370 | 0.024 | 0.931 | 0.993 |
| Head is female | -0.304 | -0.016 | 0.927 | 0.995 |
| Age of head | -0.172 | -0.018 | 0.715 | 1.020 |
| Head's age squared | -0.208 | -0.014 | 0.676 | 1.040 |
| Household has improved water source during winter | 0.139 | 0.019 | 0.978 | 0.997 |
| Household never worried about food last 4 weeks | 0.028 | -0.031 | 1.040 | 0.960 |
| Number of food insecurity months | -0.039 | 0.037 | 0.848 | 0.972 |
| Household has an outstanding debt | 0.086 | 0.023 | 1.147 | 1.036 |
| Household experienced drought/ irregular rain | 0.059 | -0.018 | 1.108 | 0.971 |
| Log total annual income from PSNP | 0.144 | 0.036 | 0.713 | 0.998 |
| Wealth index |  |  |  |  |
| 2^nd^ quartiles | -0.249 | 0.013 | 0.765 | 1.016 |
| 3^rd^ quartiles | 0.113 | 0.046 | 1.114 | 1.044 |
| 4^th^ quartiles | 0.339 | -0.045 | 1.366 | 0.968 |
| Number of ill members last month | 0.179 | -0.021 | 1.559 | 1.008 |
| Head has disability | -0.195 | -0.019 | 0.731 | 0.969 |
| CBHI understandings Index | 0.276 | -0.014 | 0.758 | 0.823 |
| CBHI perceptions Index | 0.285 | 0.007 | 0.587 | 0.986 |
| Village distance from the district capital |  |  |  |  |
| 11-20 km | -0.009 | 0.008 | 0.990 | 1.010 |
| 21-40 km | 0.081 | -0.013 | 1.013 | 0.998 |
| +40 km | -0.125 | -0.004 | 0.704 | 0.990 |
| Number of years the village has been in PSNP | 0.020 | 0.038 | 1.000 | 0.893 |
| Distance to the nearest health centre | 0.040 | -0.033 | 1.099 | 0.904 |
| Distance to the nearest health facility with a doctor | 0.034 | -0.008 | 1.082 | 1.156 |
| Nearest health facility admits people covered with CBHI | 0.048 | 0.008 | 0.862 | 0.974 |

**Appendix 5. Sensitivity analysis and robustness check**

**Sensitivity Analysis**

Following Becker and Caliendo (75), we also checked if hidden biases exist due to unobserved heterogeneity between insured and non-insured households using the Mantel and Haenszel (MH) statistic bounds for the binary outcomes (whether members sought care for illness last month, sought care from health professionals last month, and visited health facility last 12 months). Rosenbaum bounds approach was used for the continuous outcome (number of total health facility visits by all household members during the past 12 months). Results show that the odds that CBHI enrolment is determined by unobservable characteristics is not significant. Specifically, the results of sensitivity analysis show that our results are robust to sensitivity from unobserved bias until Gamma critical values of 1.6, 1.65, 1.8, and 1.6 for seeking healthcare for illness last month, seeking healthcare from skilled health professional last month, visiting a health facility for medical consultation or check-ups in the past 12 months, and the number of health facility visits by all members in the household in the past 12 months, respectively. These imply that only at 60%, 65%, 80%, and 60% increase in the odds of enrolment due to unobserved bias for aforementioned outcomes, respectively, would our results be sensitive to unobserved bias (*P*<0.05). This means that up to these levels of unobserved bias, our results are robust.

**Robustness Check**

In order to check the robustness of our results (Table A5) to estimation methods, we re-run the PSM for significant outcomes using a more restricted calliper width (0.02 compared to 0.04 in the main analyses) and using two nearest neighbour matching (compared to one-to-one matching in the main analyses). Despite slight changes in the extent of impacts, the findings were robust for reducing the calliper width by half and increasing the number of non-insured nearest neighbour households to be matched from one to two.

Table A5. Robustness check of PSM results

| ***Types of healthcare services*** | Treatment effects | | | |
| --- | --- | --- | --- | --- |
|  | ATE | | ATT | |
|  | Implementing calliper width of 0.02 | | | |
| ***Outpatient health services (N=5,377)*** | Coef. | 95% CI | Coef. | 95% CI |
| Sought care for illness last month | 0.076^***^ | [0.047,0.106] | 0.084^***^ | [0.048,0.120] |
| Sought care from health professionals last month | 0.079^***^ | [0.050,0.107] | 0.086^***^ | [0.051,0.121] |
| Visited health facility last 12 months | 0.13^***^ | [0.098,0.167] | 0.13^***^ | [0.084,0.167] |
| No. of total health facility visits last 12 months | 0.84^***^ | [0.628,1.044] | 0.86^***^ | [0.59,1.124] |
|  | Implementing a one-to-two nearest neighbour matching | | | |
| ***Outpatient health services (N=5,374)*** | Coef. | 95% CI | Coef. | 95% CI |
| Sought care for illness last month | 0.075^***^ | [0.049,0.102] | 0.084^***^ | [0.052,0.116] |
| Sought care from health professionals last month | 0.077^***^ | [0.051,0.103] | 0.087^***^ | [0.055,0.118] |
| Visited health facility last 12 months | 0.135^***^ | [0.103,0.166] | 0.136^***^ | [0.099,0.173] |
| No. of total health facility visits last 12 months | 0.830^***^ | [0.637,1.022] | 0.892^***^ | [0.648,1.136] |

95% confidence intervals in brackets; Std. Err. adjusted for clustering in village; ^*^*p*< 0.05, ^**^*p*< 0.01, ^***^*p*< 0.001

**Appendix 6. Variable types and measurements**

| **Variable** | **Type** | **Range** | **Measurement** |
| --- | --- | --- | --- |
|  | **Individual/ household head specific** | | |
| Age of head | Continuous | 15-98 | Age in completed years as reported by the respondent. |
| Head is literate | Binary | 0: No 1: Yes | Reported by the respondent. The respondent was asked “Can the head of household read and write in any language?” If the response was “*No”*, we coded educational attainment: none equal to 0, if the response was “*Yes”*, then we coded it as 1. |
| Head is married | Binary | 0: No 1: Yes | The respondent was asked “What is the head of household’s current marital status?”. Response options include: 1=Married or living together (monogamous), 2=Married or living together (polygamous), 3=Divorced or separated or deserted, 4=Widowed and 5=Never married. For regression analyses, we use a dummy variable equal to 1 if the head is currently married (options 1 and 2) and equal to 0 otherwise. |
| Head is female | Binary | 0: No 1: Yes | The sex of head of household is coded as 0 if the head is Male and 1 if the head is Female . |
| Head has a disability | Binary | 0: No 1: Yes | Disability is based on the Washington Group definition. The head was classified as having a disability if the head at least had some difficulty in seeing or hearing or remembering/concentrating or walking/climbing steps or communicating or self-care. Head was classified as having no disability if s/he had no difficulty in performing any of the above activities |
| **Household** | | | |
| Household never worried about food last 4 weeks | Binary | 0: No 1: Yes | The respondent was asked “In the **past four weeks**, did you worry that your household would not have enough food?” as a household. The responses were: Never=1, Rarely (once or twice) =2, Sometimes (3 – 10 times) =3 and Often (more than 10 times) =4. For the regression analysis, those who responded never were coded as 1 and 0 otherwise. |
| Number of food insecurity months | Continuous | 0-12 | The respondent was asked “In the past 12 months, have you been faced with a situation when you did not have enough food to feed the household?” as a household. Those who said “*Yes*” were also asked to indicate the months in which the household experienced the incident in the past 12 months. For the regression analysis, those who replied “*No*” for the first question were assigned 0 months of food insecurity and the number of months with incidents of food insecurity were counted for those who replied “*Yes*” to the first question. |
| Household has an outstanding debt | Binary | 0: No 1: Yes | As reported by the respondent for the question “Does your household have any outstanding debts to other households or institutions obtained in last 12 months (excluding purchases on credit)?” |
| Household experienced drought/ irregular rain | Binary | 0: No 1: Yes | The respondent was asked “*During the last 12 months, was your household affected negatively by* drought/ irregular rain?” as a household. For the regression analysis, the response was coded as 1 if the respondent said “*Yes*” for drought/ irregular rain and 0 otherwise. |
| Number of children aged 0-14 years | Continuous | 0-8 | The number of household members aged 14 years or below. |
| Number of adults aged 15-64 years | Continuous | 0-8 | The number of household members aged between 15 and 64 years, inclusive. |
| Number of elderly aged 65 and above years | Continuous | 0-3 | The number of household members aged 65 years and above. |
| Log total annual income from PSNP | Continuous | 0-11.019 | The respondent was asked “*How much has your household received as payment as a PSNP public work client in the last 12 months (in Birr)*?” for public work beneficiaries and “*How much has the household received from PSNP as a Direct Support beneficiary in the last 12 months (in Birr)*?” for permanent direct support clients. For the regression analysis, these values were converted to logarithm. |
| Wealth index | Categorical | 1=First quartile, 2= Second quartile, 3=Third quartile, and 4=Fourth quartile | The wealth index was constructed using Principal Component Analysis from dwelling quality indicators, total numbers of livestock in Tropical Livestock Unit, ownership of household durables and farming assets including land size. The scores were categorized in quartiles for the regression analysis. |
| Number of ill members last month | Continuous | 0-8 | The respondent was asked if each member of the household had an illness in the past month or not. This variable is a count of the number of household members who had an illness. respondent. |
| CBHI understandings Index | Continuous | -2.869 -4.701 | The understanding index was constructed based on households’ responses to whether CBHI covers medical costs related to pregnancy, CBHI fully covers certain drugs or surgery, members need to pay part of the cost even if services are covered by CBHI, premium is returned if no medical services were sought, and if enrolled households need to pay some costs in advance. We used Factor Analysis to construct the index. |
| CBHI perceptions Index | Continuous | -2.412 - 0.475 | The Perception index was formulated based on responses to two questions: 1) “*Do you believe that enrolling in CBHI will make it easier for members of your household to seek health care when needed?”* and 2) “*Do you believe that being enrolled in the CBHI plan will make health care more affordable for you and the members of your household?*” We used Factor Analysis to construct the index. |
| Household has improved water source during winter | Binary | 0: No 1: Yes | The respondent was asked “*What is the main source of drinking water for members of your household in winter?*”. The responses were: Piped water (subclassifications: Piped into dwelling, Piped into compound, yard or plot, Piped to neighbour, Public tap / standpipe or Tube Well, Borehole), Dug well (subclassifications: Protected well, Unprotected well), Protected spring, Unprotected spring, Rainwater collection, Tanker-truck (*Boti*), Cart with small tank / drum, Surface water (subclassifications: River/ stream, Dam, lake, pond, canal, irrigation channel), Bottled water and Other (specify). For the regression analysis, we considered water sources from piped water subclassifications, protected well, protected spring, rainwater collection, and bottled water as improved sources and coded as 1 and 0 otherwise. |
|  |  | **Community** | |
| Village distance from the district capital | Categorical | 1 = 11-20 km, 2 = 21-40 km, and 3 = +40 km | A knowledgeable community member was asked “*How far is the nearest woreda capital of this kebele from this kebele town/village*?”. For the regression analysis, the responses were categorized into 3 groups: 11-20 km, 21-40 km and above 40 km. |
| Number of years the village has been in PSNP | Continuous | 0-14 | A knowledgeable community member was asked “In which years has the PSNP operated in this community?”. If PSNP was introduced in the village only less than 1 year ago, it is recoded as 0. |
| Distance to the nearest health facility with a doctor | Continuous | 0-110 km | Reported by knowledgeable community member. A knowledgeable community member was asked “*What is the distance to the nearest hospital/health facility where there is a medical doctor or health officer from this kebele town/village?*” If in the village health centre or hospital has a doctor or health office, it was recorded as 0 km. |
| Distance to the nearest health center | Continuous | 0-51 km | Reported by knowledgeable community member. A knowledgeable community member was asked “*What is the distance to the nearest health centre from this kebele town/village*?”. If the health centre is in the village, it was recorded as 0 km. |
| Nearest health facility admits people covered with CBHI | Binary | 0: No 1:Yes | Reported by knowledgeable community member. A knowledgeable community member was asked “*Does the nearest health facility (post/clinic/hospital) admit people who have been covered under CBHI?*” A “*Yes*” response was coded as 1 and 0 otherwise. |

1. In order to get the SD of the logit of propensity scores, we run logit regression of CBHI enrolment on a set of covariates (presented in Table 1 of the main manuscript) and predicted the propensity scores. Then, we summarized the scores and calculated 0.2SD for the propensity scores (0.2*0.1917042=0.0383), [↑](#footnote-ref-1)
